# Disparities and Discrimination against People with HIV/AIDS by Healthcare Providers in Iran

**Published:** 2019-10

**Authors:** Ahmad KALATEH SADATI, Kamran BAGHERI LANKARANI

**Affiliations:** 1. Department of Sociology, Yazd University, Yazd, Iran; 2. Health Policy Research Center, Institute of Health, Shiraz University of Medical Sciences, Shiraz, Iran

## Dear Editor-in-Chief

HIV/AIDS stigmatization is a public health impediment (1). Stigmas create significant barriers to HIV prevention, testing, and care and can become internalized by people living with HIV/AIDS (2). An important point is that patients that hide their diagnosis is because of fear of stigma and exclusion (3). This can lead to prevalence of the disease in the society. Generally, healthcare providers are expected to know the effects of stigma and try to decreasing discrimination.

This qualitative content analysis was conducted in Shiraz, Iran in 2016. This study was supported by Shiraz HIV/AIDS (H/As) Research Center at Shiraz University of Medical Sciences. Study participants included 11 women infected with H/A and referred in an H/As-dedicated center, called Lavan. Data were collected with focus group discussions (FGDs) method. The main questions in discussions was ‘*what experiences you have ever had with HIV?*’

Results showed that all participants experienced discrimination by the society as well as health providers. They have experienced discrimination in hospitals or clinics. Three of them narrated the worst experience in the clinic. A participant with back pain referred to general physician and the doctor pushed her chair back and said ‘do not move’ to the patient. Other participants with hyperthyroidism and gynecologic problems were disrespected by their doctors, too. Because of this, these women come to a conclusion that if their diseases were revealed somewhere they were ignored. Therefore, they fear of diagnostic disclosure and they try to hide their diseases as well as possible. This finding is in line with another study that showed doctors and nurses involved with HIV patients, treat them very badly; with insult, humiliation, neglect and lack of response (4). Moreover, multidimensional stigma, rejection, and insult and discrimination in receiving health services were showed (5).

In this situation, health system in Iran needs to pay close attention to the disparities and discrimination against people living with HIV seriously. Socialization of healthcare providers especially physicians and nurses about the importance of stigma and discrimination is inevitable. In addition, health law needs to pay attention to the lost rights of these people. Since health is important rules must be in a direction that if anyone discourages these patients or refuse treatment and care he/she is being convicted.
